# In Situ IR Spectroscopy Studies of Atomic Layer-Deposited SnO_2_ on Fullerenes for Perovskite Photovoltaics

**DOI:** 10.1021/acsami.4c09630

**Published:** 2024-10-16

**Authors:** Andrea E. A. Bracesco, Joost van Himste, Wilhelmus M. M. Kessels, Valerio Zardetto, Mariadriana Creatore

**Affiliations:** † Plasma & Materials Processing, Department of Applied Physics and Science Education, Eindhoven University of Technology (TU/e), P.O. Box 513, 5600 MB Eindhoven, The Netherlands; ‡ TNO-partner in Solliance, High Tech Campus 21, 5656 AE Eindhoven, The Netherlands; § Eindhoven Institute of Renewable Energy Systems (EIRES), 5600 MB Eindhoven, The Netherlands

**Keywords:** perovskite PV, atomic layer deposition, SnO_2_, infrared spectroscopy, fullerenes, interfaces

## Abstract

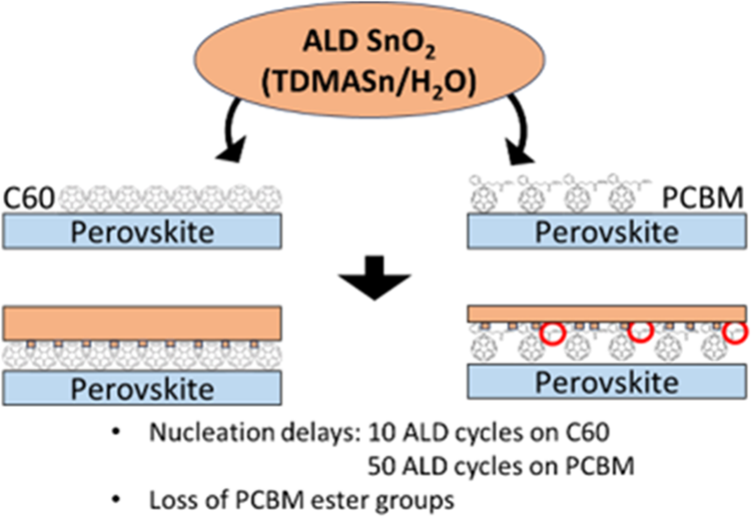

In recent years, atomic layer deposition (ALD) has established itself as the state-of-the-art technique for the deposition of SnO_2_ buffer layers grown between the fullerene electron transport layer (ETL) and the ITO top electrode in metal halide perovskite-based photovoltaics. The SnO_2_ layer shields the underlying layers, i.e., the fullerene-derivative materials such as C60 and PCBM, as well as the perovskite absorber, from water ingress and damage induced by the sputtering of the transparent front contact. Our study undertakes a comprehensive investigation of the impact of SnO_2_ ALD processing on fullerenes by means of *in situ* spectroscopic ellipsometry (SE) and transmission infrared spectroscopy (FTIR). While no difference in SnO_2_ bulk properties is observed and the perovskite absorber degradation is nearly entirely avoided during exposure to heat and vacuum, when the absorber is introduced beneath the organic ETLs, a SnO_2_ growth delay of about 50 ALD cycles is measured on PCBM, whereas the delay is limited to 10 cycles in the case of growth on C60. Notably, FTIR measurements show that while C60 remains chemically unaffected during SnO_2_ ALD growth, PCBM undergoes chemical modification, specifically of its ester groups. The onset of these modifications corresponds with the detection of the onset, after the initial delay, of ALD SnO_2_ growth. It is expected that the modification that the PCBM layer undergoes upon ALD SnO_2_ processing is responsible for the systematic lower photovoltaic device performance in the case of PCBM-based devices, with respect to C60-based devices.

## 1 Introduction

Inverted semitransparent perovskite solar cells (PSCs) are the subject of extensive research for both single junction and tandem applications. In both cases, a sputtered top ITO electrode is introduced to enable illumination from the top of the device. However, direct ITO sputtering leads to perovskite and fullerene damage. Therefore, a protective, dense, and conformal layer such as SnO_2_,^1^ processed by ALD, serves as a buffer layer. Moreover, its implementation in the PSC structure imparts thermal and environmental stability to the device.^2−4^ The commonly used electron transport layers in this configuration are (C60-Ih)[5,6]fullerene (C60), and [6,6]-phenyl-C61-butyric acid methyl ester (PCBM).

Ideally, an ALD process requires surface functional groups, such as hydroxyl groups or amines, to promote ALD precursor chemisorption.^5^ For PCBM, the ester functionality may serve as nucleation site. However, for C60, no functional group is present, potentially making it a chemically challenging substrate for film growth. When looking into literature, only few studies on ALD growth on fullerenes were found. Yu et al. investigated the growth of ZnO and Al-doped ZnO (AZO) on PCBM. They indicated that the substrate is unreactive toward exposure to both diethyl-zinc (DEZ) and water.^6^ This is because DEZ, which is a weak Lewis acid, only physisorbs on the surface. and no growth occurs.^7^ If instead trimethyl-aluminum (TMA) is adopted, Al_2_O_3_ growth occurs on PCBM, primarily via TMA chemisorption on the ester group, because of the TMA strong Lewis acid character.^8−12^ Specifically, it was reported that TMA forms a covalent bond between the Al metal center and the carboxylic moiety, forming the adduct −C–O-Al-(CH_3_)_2_.^6^ The subsequent H_2_O dosing step reacts with the methyl ligands providing the hydroxyl groups which enable further growth of the layer. Similar conclusions were reached by Gong et al. and Parsons et al. for the growth of Al_2_O_3_ ALD layers on a wide range of polymers.^8,13^ More recently, Wang et al. successfully adopted surface functionalization strategies. Specifically, 3-aminopropyl triethoxysilane (APTS), or 3-mercaptopropyl triethoxysilane (MTPS), were added to the PCBM solution to provide amino- and silanol- surface groups for the chemisorption of TDMA-Sn, i.e., the Sn-precursor for SnO_2_ growth.^14^ In the case of C60, Raiford et al. reported on the limited growth of SnO_2_ during the early stages of growth (within the first 10 ALD cycles). Similar results were also reported for ALD SnO_2_ growth on PCBM, suggesting that the ester groups present on PCBM do not necessarily lead to a more favorable growth. Functionalization of C60 through ethoxylated polyethylenimine (PEIE),^15^ promoted SnO_2_ growth. More recently, Palmstrom et al., adopted the same strategy to grow AZO on C60/PEIE.^16^

Based on this literature overview, it can be concluded that the issues of ALD nucleation and film growth on chemically challenging substrates like the fullerenes here under investigation, are in general addressed by adding functionalities to their surface to promote the ALD precursor chemisorption. We believe that the understanding of SnO_2_ ALD nucleation and film growth on fullerene layers with different chemistries can eventually lead to better engineering of perovskite devices. In the present study we systematically investigate the fullerene exposure to the ALD SnO_2_ precursor/coreactant, namely TMDA-Sn and H_2_O, and to full ALD cycles, with and without the presence of an underlying perovskite absorber beneath the fullerene ETLs. We address the nucleation and growth of SnO_2_ on PCBM and C60 by means of *in situ* diagnostics, namely spectroscopic ellipsometry and transmission IR spectroscopy, and correlate the results with the performance of perovskite solar cells with ALD SnO_2_ on PCBM and C60. We report the detection of a delay of 50 ALD cycles in SnO_2_ growth on PCBM, which is five times longer than that observed on C60. In parallel, the growth on the two fullerene ETLs is found to have a negligible influence on the bulk material properties of the ALD layers. Furthermore, our observations show that the underlying perovskite is efficiently protected from direct interaction with the ALD chemistry preserving its composition. We show that the adoption of C60 leads to higher open-circuit voltage and fill factor, resulting in a 2.9% (absolute value) higher power conversion efficiency when compared to PCBM-based devices. We argue that this difference in device performance can be attributed to detected PCBM surface modification occurring during ALD SnO_2_ growth, namely a loss of the C=O vibration modes of PCBM as evidenced by IR spectroscopy. The C60 surface is, instead, unaffected by SnO_2_ growth.

## 2 Experimental Section

### 2.1 Perovskite Solar Cell Fabrication

The characterization of the ALD layers was conducted on B-doped, double-side polished crystalline silicon (100) substrates with a thickness of 400 μm and a resistivity of 10–20 Ohm-cm. For the PSCs devices, patterned glass/ITO substrates with a resistivity of 13–15 Ω/sq from Naranjo Substrates were ultrasonically cleaned with soap water, deionized water, and isopropanol. This was followed by a UV-ozone treatment for 30 min. A perovskite film of Cs_0.15_FA_0.85_Pb(I_0.92_Br_0.08_)_3_ was deposited on one side of either the crystalline silicon substrates or the patterned glass/ITO substrates. Before the perovskite deposition, a poly(triarylamine) (PTAA) solution in toluene 2 mg/mL from Sigma-Aldrich was spin-coated at 5000 rpm for 30 s and annealed at 100 °C for 10 min. The perovskite solution was prepared by mixing a 1.33 M concentration of the following precursors: PbBr_2_ (99.9%) and PbI_2_ (99.999%), both from TCI; FAI (99.9%) and FABr (99.9%) both from Greatcell (Dyesol); and CsI (99%, Sigma-Aldrich), in anhydrous DMF:DMSO (vol. ratio = 9:1), followed by stirring overnight at room temperature. Inside an N_2_-filled glovebox, the perovskite solution was then spin-coated with a two-step procedure: 10 s at 200 rpm and then 30 s at 5000 rpm. Ten s into the second step, 300 mL of chlorobenzene is poured on the spinning substrate. The resulting film was then annealed on a hot plate at 100 °C for 10 min, resulting in a perovskite film with a thickness of about 450 nm. For devices, or samples, with a PCBM interlayer, a 20 mg/mL PCBM solution in chlorobenzene, from Solenne B.V. (99%), was spin-coated onto the perovskite layer at 1500 rpm for 50 s. For devices with a C60 interlayer, 20 nm were thermally evaporated. Next, SnO_2_ layers were grown either directly on the c-Si substrates or on the perovskite decorated with either PCBM or C60. 400 ALD cycles were carried out, corresponding to an expected thickness of 44–48 nm. Afterward, 180 nm of ITO was sputtered at room temperature by a RF magnetron sputtering with a power of 54 W in AJA International system, from a 90% In_2_O_3_ – 10% SnO_2_ targe. On top of the ITO, 100 nm of Ag, acting as the top electrode, was thermally evaporated. For the reference device, a bathocuproine (BCP) solution in ethanol (0.5 mg/mL) was spin-coated at 4000 rpm for 40 s on top of PCBM, prior to the evaporation of the top electrode. 100 nm of Cu was thermally evaporated at a pressure of 10^–6^ mbar. A shadow mask was used for electrode deposition in all cases.

### 2.2 Device Characterization

The electrical characterization of the perovskite solar cells was performed under a solar simulator calibrated to AM 1.5 using a commercial c-Si reference cell (ReRa Solutions, IEC 60904–9 compliant and certified) and using a Keithley 2400 source meter to collect the current-voltage (J-V) curves and to determine the maximum power point. A shadow mask with a designated cell area of 0.09 cm^2^, was used during the measurements. The J-V curves were measured in both scan directions using a Keithley 2400: (i) reverse scan from 1.2 to −0.2 V and (ii) forward scan from −0.2 to 1.2 V, and the scan rate is kept at 200 mV/s. After the J-V scans, the PCE deduced from the maximum power point measurement was tracked for 120 s. Multiple PSCs were measured for each device structure to obtain sufficient statistics. The light intensity of the source is calibrated to 100 mW/cm^2^ with a silicon reference cell to simulate the AM1.5 spectrum.

### 2.3 Atomic Layer Deposition of SnO_2_

Atomic layer-deposited (ALD) SnO_2_ films were prepared using tetrakis(dimethylamido)-Sn(IV) (TDMA-Sn), 99.9% from STREM Chemicals, as the metal–organic precursor and water as the co-reactant. The precursor was kept at 50 °C and supplied to the ALD chamber, kept at 100 °C and at a base pressure of 10^–5^ mbar, in vapor-drawn mode, with an ALD cycle consisting of a 500 ms TDMA-Sn dose, followed by a purge step of 5 s, then a 50 ms H_2_O vapor dose, followed by a purge step of 10 s. The ALD SnO_2_ depositions for the perovskite solar cell devices were carried out in an Oxford Instruments FlexAL ALD reactor. The *in situ* FTIR characterization during ALD processing was carried out, under the same conditions as those selected for FlexAL, in a home-built reactor.^17,18^

### 2.4 In Situ Spectroscopic Ellipsometry

The thickness evolution of the ALD SnO_2_ films on c-Si substrates was determined via *in situ* spectroscopic ellipsometry (SE), using a J.A. Woollam, Inc. NIR M2000U ellipsometer. A M2000D rotating compensator ellipsometer from J.A. Woollam Inc. was instead used for *ex situ* measurement and employs a variable angle SE (VASE) from a spectral range of 1.25 to 6.5 eV, between 65° and 75°. The calculated growth per cycle (GPC) is 0.12 ± 0.01 nm/cycle. The SnO_2_ layers were fitted using the Cauchy formula with an Urbach tail absorption due to its bandgap being about 3.6 eV. For the ALD growth on fullerenes, the PCBM and C60 layers, having both a bandgap of about 2.0 eV, were modeled using the Cauchy formula with an Urbach tail absorption in the 1.2 to 2.7 eV range. To extend the fitting range, their Cauchy-Urbach tail was converted into a B-spline, which extends the fit up to 5 eV. The measured ψ and Δ, together with fit parameters and fitted curves are shown in the Supporting Information, Figure S1 and Table S1 in the Supporting Information. In parallel, UV–vis measurements were carried out, with a PerkinElmer Lambda 1050 UV–vis-NIR spectrophotometer, to determine the absorption coefficient, used to determine the optical bandgap of the ALD SnO_2_ layers.

### 2.5 Infrared Spectroscopy

The *in situ* IR measurements were performed using a Bruker Vector 22 FTIR spectrometer with a mid-infrared light source (Globar 10,000–50 cm^–1^) and a liquid N_2_-cooled Bruker MCT 313 detector.^19,20^ KBr windows were mounted on flanges connected to the ALD reactor chamber on both the source and detector sides of the reactor. The samples were placed vertically with respect to the IR beam in the ALD reactor using a 4-axis manipulator (PREVAC). A schematic representation of the setup can be found in a previous publication.^18^ The reactor was pumped down to a base pressure of 10^–5^ mbar, corresponding to a waiting time of 30 min before the start of the IR measurements, and the samples were exposed to vacuum and heat conditions for a maximum of 3 h. The IR measurements consisted of 4096 consecutive averaged absorbance scans with a resolution of 8 cm^–1^. These absorbance spectra were measured every 10 min. The results are presented as differential absorbance spectra, obtained by subtracting the pristine perovskite spectrum from the absorbance of the sample after the modifications or subtracting subsequent spectra from each other. Negative spectral features correspond to the removal of species from the surface or the bulk of the sample. Positive features instead are related to the addition of new species. Baseline corrections were performed using Origin by fitting a B-spline and subtracting it from the measured absorbance. Integrated areas of IR bands of interest were calculated using the built-in procedure in Origin and normalized to the one corresponding to that of a 450 nm thick pristine perovskite, as such they provide relative values. In situ Fourier transform infrared spectroscopy (FTIR) measurements were carried out during ALD SnO_2_ deposition, on top of the perovskite/fullerene-based ETLs, every 10 ALD cycles.

### 2.6 X-ray Photoelectron Spectroscopy

The surface composition, given as atomic percent, of the ALD SnO_2_ grown on c-Si and on fullerene-based ETLs was investigated by means of XPS in a Thermo Scientific K-Alpha system, equipped with an Al Kα X-ray source (1487 eV) without any presputtering step. Using the THERMO AVANTAGE software, each spectral feature was fitted with a convolution of Gaussian/Lorentzian mixed peaks, which have a fixed full-width half-maximum for each chemical state across all the samples. For each sample, the binding energies (BE) of the spectral features are provided with respect to that of the C–C/C-H spectral feature of adventitious carbon centered at 284.8 eV. Depth profiles were also recorded by performing repeated sputtering steps using the built-in Argon ion gun with an ion energy of 500 eV.

### 2.7 X-ray Reflectivity

XRR measurements, to determine the density of ALD SnO_2_ layers, were performed on a Bruker D8 DISCOVER, equipped with a Cu k-α source (1.5406 Å) and analyzed with the LEPTOS software.

### 2.8 Rutherford Backscattering Spectroscopy and Elastic Recoil Detection

The samples were analyzed by means of Elastic Recoil Detection (ERD) and Rutherford Backscattering Spectrometry (RBS) to determine the composition and areal density of the films. The measurements were carried out by Detect99 using the HVE Singletron located at the DIFFER Institute in Eindhoven.^21^ A 2000 keV He^+^ beam is employed. ERD was performed with a 75° sample tilt and the detector at a recoil angle of 19.3°. RBS was performed with perpendicular incidence and two detectors at scattering angles of 170 and 110°. A glassy carbon substrate was used (instead of c-Si), to avoid overlapping contributions originating from substrate features under the C, N, and O peaks in the RBS spectra. An intermediate Al_2_O_3_ layer was deposited to separate the spectral carbon feature of the substrate from the carbon peaks of the relevant layers. The WiNDF simulation package was used for all simulations to determine the absolute concentration, given as at./nm^2^.^22^ To determine the film density, the thickness of the SnO_2_ layers grown on the glassy carbon/Al_2_O_3_/fullerene was estimated by calibrating the sputtering time during XPS depth profiling of SnO_2_ grown on c-Si.

### 2.9 Transmission Electron Microscopy

Cross-sectional TEM lamellas were prepared using a Focused Ion Beam (FIB) lift-out procedure. The TEM studies were carried out using a probe-corrected JEOL ARM 200F, operated at 200 kV, equipped with a 100 mm^2^ Centurio SDD EDX detector.

## 3 Results and Discussion

### 3.1 PSC Device Performance

**Figure 1:**
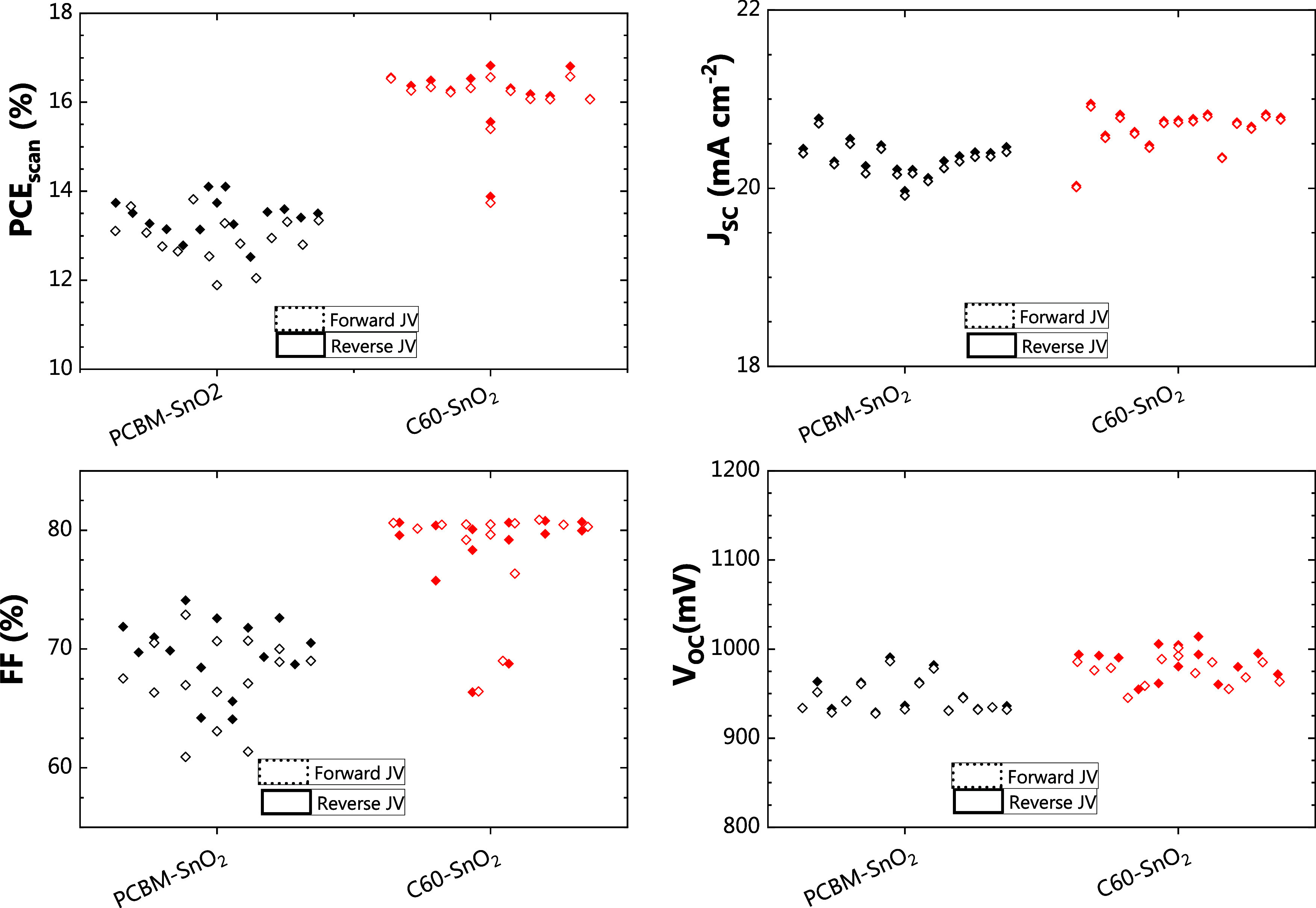
PCE, *J*_SC_, FF, and *V*_OC_ of the devices employing two different ETL configurations, PCBM/SnO_2_ and C60/SnO_2_. 400 ALD SnO_2_ cycles are carried out, corresponding to an expected thickness of 44–48 nm, based on SnO_2_ growth on c-Si.

Figure 1 presents the JV characteristics of semitransparent perovskite solar cells employing SnO_2_ with either C60 or PCBM serving as ETLs, in combination with an ALD SnO_2_ buffer layer. The PCBM/SnO_2_ devices exhibit a PCE of 13.2 ± 0.2% which is lower than the 16.2 ± 0.1% of the C60/SnO_2_ counterpart. The devices using PCBM/SnO_2_ have on average a 10% absolute value lower FF and a 50 mV lower *V*_OC_ than the C60-based devices. The differences in these two parameters suggest that charge extraction and possibly interface recombination may be taking place in the case of PCBM/SnO_2_. An opaque device, serving as a reference to highlight the quality of the perovskite absorber, is reported in Figure S2. It adopts an organic ETL bilayer consisting of PCBM and BCP (bathocuproine) and shows an efficiency of 18.3 ± 0.3%, in line with values reported in literature.^23,24^ The JV curves of the champion devices of each ETL configuration are reported in Figures S3a and Table SII and show negligible hysteresis and no s-shape. This indicates that the SnO_2_ thickness (estimated between 44 and 48 nm, based on the GPC of SnO_2_ measured on c-Si) is sufficient to protect the perovskite and fullerene layers from ITO sputtering damage. Vice versa, as shown in Figure S3b, an excessively thin SnO_2_ buffer layer would result in a significantly s-shaped JV curve. A detailed discussion on the measured thickness of the SnO_2_ layers, and their growth on the different ETLs, can be found in Section 3.2. The following sections of the manuscript investigate the potential reasons behind the difference in performance between the two devices, by addressing the growth of SnO_2_ on fullerenes via *in situ* diagnostics and characterization of the film properties.

### 3.2 SnO_2_ Layer Properties

**Figure 2:**
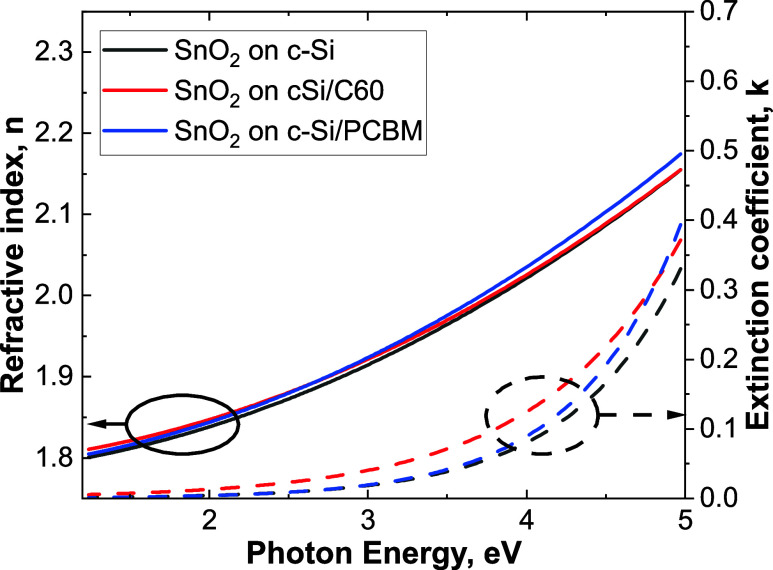
Refractive index and extinction coefficient of 48 nm SnO_2_ layers grown on c-Si and c-Si/PCBM or C60.

Figure 2 shows the refractive index and extinction coefficient values of the SnO_2_ layers grown on c-Si, c-Si/C60, and c-Si/PCBM. No noticeable difference can be detected, suggesting that the optical properties of bulk SnO_2_ are independent of the growth substrate. At 1.96 eV, the refractive index is 1.84 (±0.02), a value consistent with that reported for 100 °C PE-ALD SnO_2_ (1.86) by Kuang et al. and for thermal ALD SnO_2_ (1.85) by Mullings et al.^25,26^ To the best of the authors’ knowledge, this is the first time that optical properties of ALD SnO_2_ grown on fullerenes are reported and inferred by *in situ* measurements. Additionally, a bandgap of about 3.6 eV,^27,28^ was calculated from the absorption coefficient measured by UV–vis on glass/ITO substrates, as shown in Figure S4.

In parallel, XPS and RBS measurements, reported in Tables SIII and SIV, indicated that the chemical composition and mass density of SnO_2_ films are also not influenced by the substrate. The measured densities of the SnO_2_ layers are 4.6 (±0.4) g/cm^3^, and 4.3 (±0.6) g/cm^3^ for SnO_2_ grown on C60 and PCBM, respectively. These values are in line with literature-reported SnO_2_ ALD layers grown using a variety of precursors and co-reactants at deposition temperatures similar to the one used in this investigation.^29−31^ At such growth temperatures, below 200 °C, ALD SnO_2_ layers are amorphous and incorporate Sn-precursor ligands, as can be seen from the inclusion of C and N species, Table SIII.^18,25,32^ The next section delves into how SnO_2_ grows on the organic ETLs.

### 3.3 Growth Delay of SnO_2_ on Fullerenes

**Figure 3:**
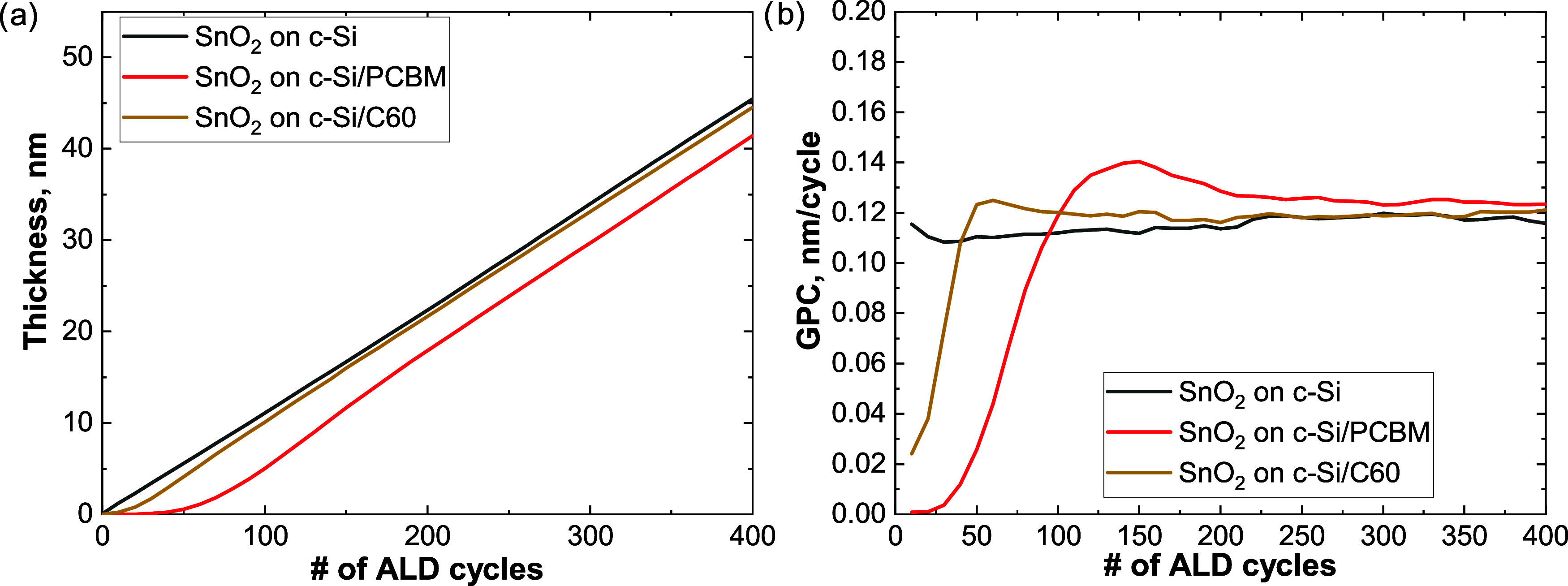
(a) Thickness evolution of SnO_2_ grown on c-Si, C60, and PCBM, measured by in situ SE (b) GPC of the SnO_2_ layers on c-Si, C60 and PCBM, inferred by the data in Figure 3a.

Figure 3a reports the film thickness evolution on the different substrates. The calculated GPC on c-Si is 0.12 nm/cycle and agrees with values reported at this temperature.^26^ The growth on PCBM and C60, shows a delay of about 48 (±3) and 10 (±3) ALD cycles, respectively. Afterward, the thickness increase proceeds linearly with a GPC of about 0.12 nm/cycle. The calculated GPC trend, shown in Figure 3b, resembles that corresponding to the well-known effect of substrate-inhibited film growth,^33^ where island growth occurs on a chemically unreactive substrate. Once the GPC reaches the steady state, layer-by-layer growth occurs.^33^ In our case, film closure would occur at a cycle number above 50 and 150 ALD cycles, on C60 and PCBM, respectively.

Based on the evidence reported above, we speculate that given the lack of chemisorption sites on C60, SnO_2_ nucleates either on defects or at previously physisorbed water at the (sub-)surface of C60, with a mechanism similar to that reported by Vervuurt et al.^34−36^ For PCBM, instead, given the larger growth delay with respect to C60, it appears that the ester groups are not promoting TDMA-Sn chemisorption and/or the defect density is lower than on C60.

### 3.4 Effect of ALD Processing Conditions on the Perovskite/Fullerene Bilayer

**Figure 4:**
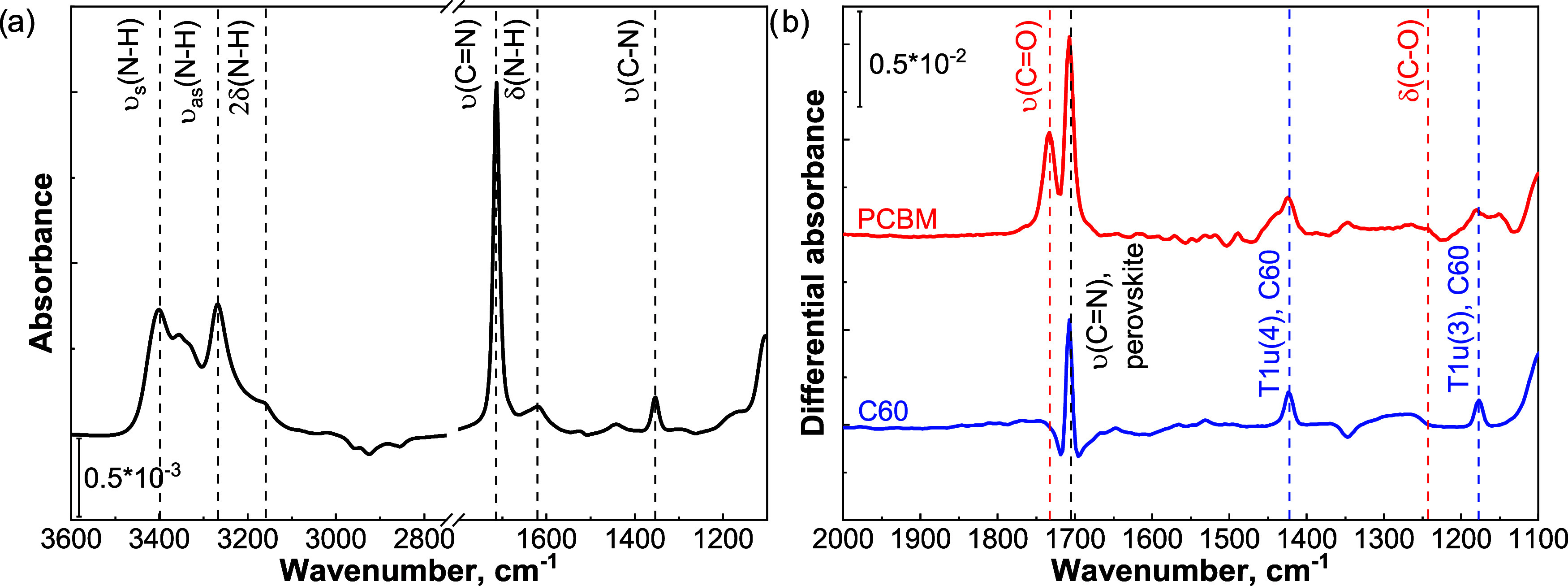
(a) Pristine perovskite absorbance spectrum measured at a reactor temperature of 100 °C and pressure of 10^–5^ mbar. The main vibrational features of the formamidinium cation (FA^+^) are assigned. (b) Differential absorbance spectra, determined by subtracting the absorbance of the pristine perovskite (prior to the application of the fullerene layer) from the absorbance of the perovskite/fullerene bilayer. The assignments of the modes belonging to the fullerene layers are reported. To be noted, the negative fringes, present on the sides of the 1713 cm^–1^ peak and at 1353 cm^–1^ of the C60 spectrum, are caused by small variations in the thickness of the perovskite absorber, between the reference and the perovskite/C60 samples.

To shed light on the change in chemistry of PCBM and C60 upon interaction with the ALD precursor and co-reactant, we adopt *in situ* IR spectroscopy. Additionally, in this investigation, we include the effect of ALD processing parameters, such as substrate temperature and vacuum level in the reactor chamber, to highlight the beneficial role of the fullerene layer in preventing damage to the underlying perovskite absorber. Figure 4a shows the FTIR spectrum of the pristine perovskite absorber. Figure 4b shows the differential spectra of the perovskite/PCBM and perovskite/C60 samples, from which the pristine perovskite spectrum has been subtracted, to discern the vibrational modes of the fullerene layers. A summary of the detected modes is provided in Table 1.

**Table 1:** Summary of IR Modes of Perovskite^32^ and Fullerene-Based ETLs in the 3500–1000 cm^–1^ Range

perovskite (formamidinium-related)^32,37−40^	PCBM^40−43^	C60^40−43^
3400–3267 cm^–1^		
N–H stretching		
1713 cm^–1^	1738 cm^–1^	
C=N stretching	C=O stretching	
1619 cm^–1^		
N–H bending		
1353 cm^–1^	1429 cm^–1^	1429 cm^–1^
C–N stretching	T1u(4)	T1u(4)
	1246 cm^–1^	
	C–O bending	
	1182 cm^–1^	1182 cm^–1^
	T1u(3)	T1u(3)

**Figure 5:**
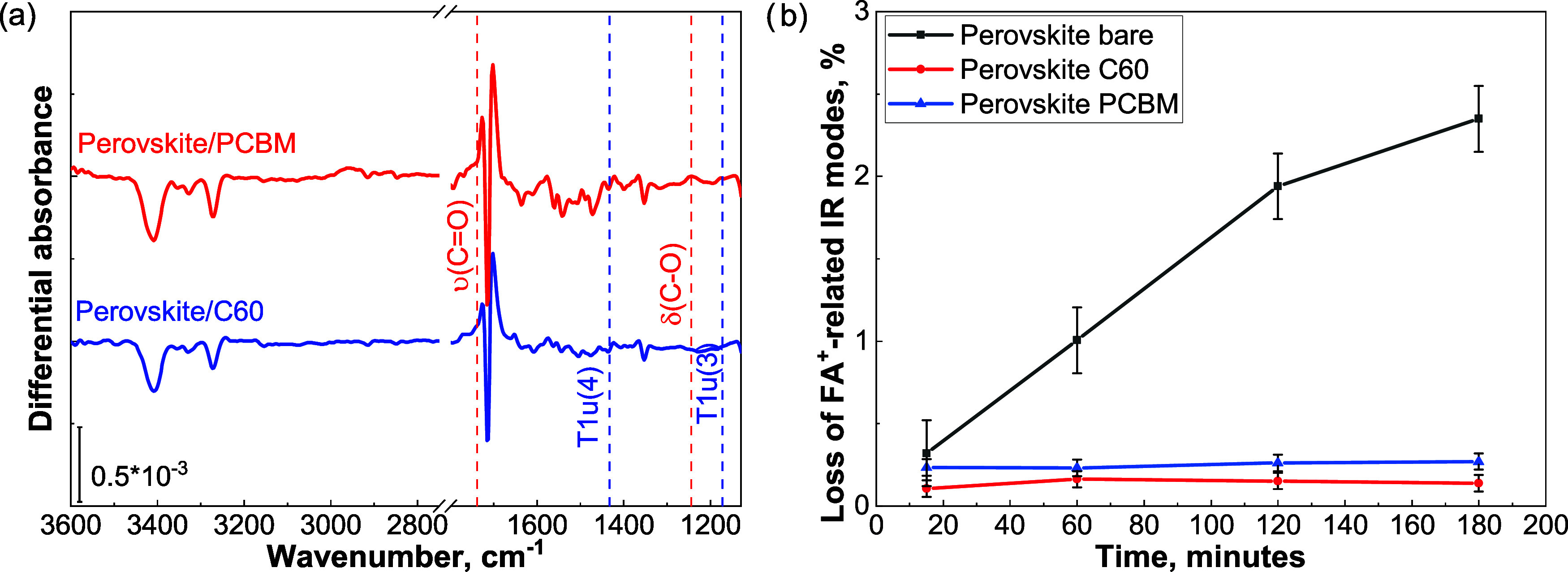
(a) Differential absorbance spectra of the perovskite/C60 and perovskite/PCBM substrates measured after 3 h of exposure to 100 °C and 10^–5^ mbar, from which the pristine perovskite spectrum shown in Figure 4a has been subtracted. For clarity, only the fullerene-related IR modes are indicated. (b) Calculated FA^+^ losses, from the integrated area of the N–H stretching region 3500 to 3000 cm^–1^.

Figure 5a shows the effect of a 3 h exposure of the perovskite/fullerene substrates to ALD processing conditions (without exposing the substrates to the ALD chemistry). The differential absorbance spectrum does not show any fullerene-related negative features, indicating that temperature and vacuum do not affect the fullerene chemistry. These findings align with reported studies which showed that fullerenes exhibit stability at temperatures exceeding 250 °C.^44−49^ The positive/negative features, centered at about 1613 cm^–1^, are instead due to the deprotonation of FA^+^.^32^

Instead, negative features in the range of 3400 to 3267 cm^–1^, are evident in Figure 5a, and are consistent with the vibrational frequencies of FA^+^ cations. A similar observation can be made for the negative features associated with the C=N stretching and C–N stretching modes centered at 1713 and 1353 cm^–1^, respectively. The positive feature at about 1702 and 1720 cm^–1^ corresponds to the deprotonation of FA^+^ into FA and the subsequent relaxation of the frequency of the C=N stretching mode.^32^Figure 5b reports the calculated integrated area of the stretching modes present between 3500 and 3000 cm^–1^, measured during the extended exposure to heat and vacuum and compared with the bare perovskite case. The presence of C60 and PCBM suppresses the loss of the organic fraction of the perovskite absorber. After 3 h, the pristine perovskite exhibits a loss of 2.3 (±0.2) %, whereas this is only 0.2 (±0.2) % and 0.3 (±0.2) % for C60 and PCBM, respectively.^50−54^ The estimated losses belonging to the bare perovskite match the values reported in a previous publication.^32^ This comparison allows us to conclude that both fullerenes exhibit good barrier properties against perovskite decomposition under vacuum and 100 °C.

### 3.5 Effect of ALD Processing on the Perovskite/Fullerene Bilayer

**Figure 6:**
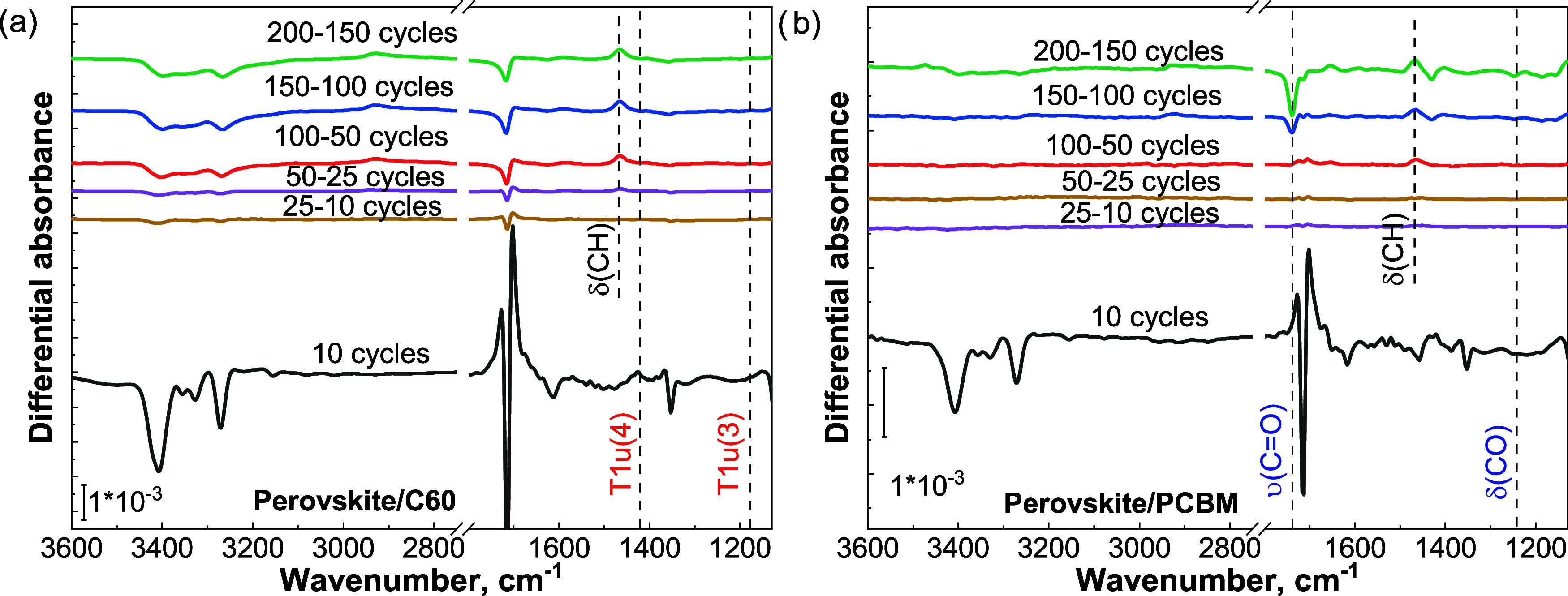
(a) Differential absorbance spectra of (a) perovskite/C60 and (b) perovskite/PCBM exposed to subsequent numbers of ALD cycles (10–200). The unassigned features correspond to vibrational modes belonging to the underlying perovskite.

To study the effect of the exposure to ALD chemistry, first we investigate how the perovskite/fullerene bilayers respond to the exposure, separately, to multiple ALD precursor or coreactant doses (i.e., no growth occurs), as reported in Figure S5a,b. No modifications to C60 or PCBM are detected. Next, we investigate the role of ALD chemistry, in its full cycle fashion, on the stability and/or decomposition of both fullerenes. Figure 6a,b show the effect of SnO_2_ growth on both systems. The spectra reveal features corresponding to perovskite degradation in the N–H region and the C=N stretching region of both perovskite/fullerene systems. Interestingly, the losses associated with the perovskite take place primarily during the first 10 ALD cycles in both cases, i.e., when the layer has not reached yet closure. This is an additional confirmation, following the results reported in Figure 5b, that the presence of the fullerene ETLs prevents damage to the underlying perovskite, by physically separating the surface of the absorber from the ALD chemistry.

No chemical modification is detected in the spectrum of C60 in Figure 6a. In the case of PCBM, a negative peak at 1738 cm^–1^, characteristic of the C=O stretching of the ester group, emerges during the first 100–150 ALD cycles. In parallel, the C–O bending mode at 1246 cm^–1^ also develops as a negative feature, although with a very weak peak intensity. These two findings suggest that the ester group of PCBM is affected when exposed to both TDMA-Sn and H_2_O.

To confirm the assignment of the detected modifications on PCBM and prove the stability of C60, Figures S6a and S6b report the effects of an extended ALD process on samples that did not include the perovskite absorber but consisted of the c-Si/fullerene structure. The results show the lack of negative features in the spectra of C60, thus confirming the chemical stability of the molecule against exposure to the ALD chemistry. Differently from this, and in agreement with the results shown in Figure 6b, PCBM presents negative features in correspondence with its C=O stretching and C–O bending modes, confirming that the ester group is modified, or abstracted. These results indicate that the presence of the perovskite absorber does not influence the chemical stability of the fullerene ETLs.

The growth of the SnO_2_ film on the fullerene layers can be inferred from indications found in Figure 6a,b, where the positive feature centered at 1466 cm^–1^ is associated with the incorporation of the Sn-precursor ligands, -N(CH_3_)_2_. This feature becomes visible between 25 and 50 cycles for SnO_2_ grown on C60. In the case of PCBM, the feature becomes visible only between 50 and 100 ALD cycles. Figure S7 provides further confirmation of this delay by comparing the integrated area of the 1466 cm^–1^ feature. The positive/negative features, centered at about 1613 cm^–1^, are instead due to the deprotonation of FA^+^.^32^

**Figure 7:**
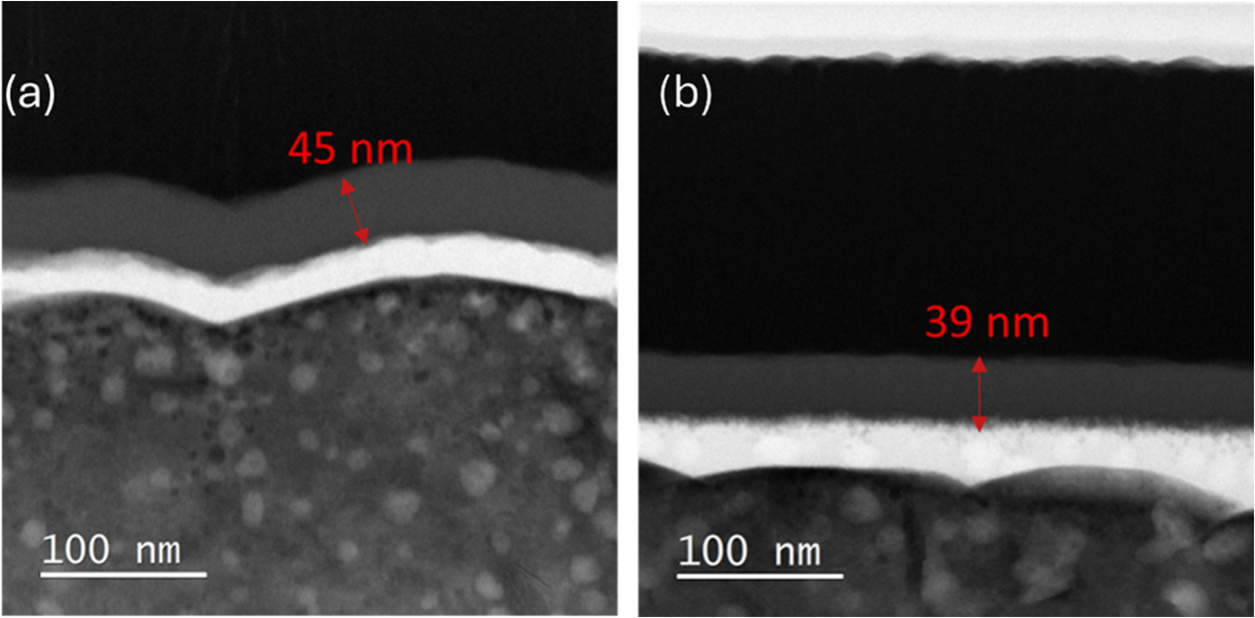
Cross-sectional TEM images of (a) perovskite/C60/ALD SnO_2_ and (b) perovskite/PCBM/ALD SnO_2_. The measured geometrical thickness of the SnO_2_ layers is reported in the images.

In parallel, cross-sectional TEM images, shown in Figure 7, were acquired to visualize and confirm the extent of the growth delay of ALD SnO_2_ on the two fullerene ETLs. As it can be seen, the measured SnO_2_ thickness on C60 is 45 ± 1 nm, while on PCBM it is 39 ± 1 nm. These values closely match with the thickness measured by *in situ* by SE during the ALD SnO_2_ growth on the cSi/fullerene systems. To be noted, the calculated growth delay for the PCBM/SnO_2_ case is estimated at about 6 nm. This value falls in the lower range of the 50 to 100 ALD cycles delay as estimated by the *in situ* FTIR measurements of the fullerene/perovskite systems. This difference can be explained in terms of lower sensitivity of the FTIR measurement. In conclusion, the SnO_2_ film growth delay is larger on PCBM than on C60.

To conclude, the detected chemical modification at the ester functionality of PCBM, occurs simultaneously with the onset of SnO_2_ growth. When reflecting upon the literature overview in the Introduction, i.e., on the reactivity of PCBM toward chemisorption of TMA and DEZ ALD precursors, it can be argued that TDMA-Sn does behave neither as acid nor as base, since the Sn metal center cannot donate or accept electrons due to the lack of lone pairs or empty orbitals. Thus, the ester functionality in PCBM is inert, or weakly active, as a chemisorption site for TDMA-Sn, thereby explaining the extended SnO_2_ growth delay on PCBM. Given that for C60 no chemical modifications are detected, we suggest that the modifications which PCBM undergoes upon SnO_2_ growth, are responsible for the loss of photovoltaic performance observed in devices employing PCBM. Interestingly, we found the same difference in device performance when spatial-ALD SnO_2_ processing is carried out on C60 and PCBM (Figure S8). The former present on average a 50 mV higher *V*_OC_, >10% higher FF, and a 4.6% higher PCE than those based on PCBM. These values are very close to those presented in Figure 1. We have also found that the measured thickness evolution of the s-ALD SnO_2_ presents a larger nucleation delay in the case of PCBM, compared to C60 (Figure S9). Similar results between the ALD- and s-ALD-based devices point out that the difference in device performance, with respect to C60, is to be solely attributed to the chemical modification of PCBM and delay in SnO_2_ growth.

## 4 Conclusions

In summary, PSCs employing either a C60 or PCBM ETL in combination with an ALD SnO_2_ buffer layer revealed differences in fill factor, open-circuit voltage, and overall performance, with C60-based devices performing better than the PCBM-based devices. Therefore, we have investigated the growth of SnO_2_ on the fullerenes, including the effect of ALD processing parameters, such as substrate temperature, vacuum level, and exposure to precursor and co-reactant doses.

The results demonstrated that the material properties of SnO_2_, such as composition, density, and refractive index are unaffected by the choice of the fullerene. However, a substrate-inhibited growth mode was detected for both C60 and PCBM, opposite to c-Si. In the case of PCBM, a delay of approximately 50 ALD cycles was observed by *in situ* spectroscopic ellipsometry, suggesting a limited presence of nucleation sites on PCBM. Instead, a delay of only 10 ALD cycles was observed for C60 case.

IR spectroscopy was adopted to investigate the chemical modifications occurring on PCBM and C60 upon temperature, half-ALD cycles, and full SnO_2_ growth cycles. The ALD growth occurred with delay on PCBM, confirming the *in situ* SE studies. Moreover, the loss of vibrational features associated with the ester group in PCBM was observed. In contrast, C60 chemistry remained unaffected and SnO_2_ growth occurred earlier than on PCBM. We concluded that the modifications detected on PCBM are responsible for the loss of performance in PSCs devices and attributed to changes in the charge extraction efficiency of the ETL/SnO_2_ interface, which leads to the decrease in *V*_OC_ and FF.

Another aspect that was investigated was the barrier properties of the fullerene layers against the absorber decomposition during ALD processing. Overall, regardless of the choice of fullerene, FA^+^-related losses, were detected. These are found to be equal to 0.25% in the presence of C60 or PCBM. In contrast, in the case of the pristine perovskite, approximately 2.3% of the total FA^+^ content is abstracted during a 3 h exposure to heat and vacuum. This finding indicates that the fullerenes effectively shield the underlying perovskite from thermal decomposition and ALD chemistry. These insights contribute to a better understanding of the chemical interaction between SnO_2_ ALD chemistry and fullerene-based ETLs, elucidating the effect of SnO_2_ growth on the loss of device performance. Moreover, they are valuable for the development of ALD processes aimed at preserving the properties of fullerene-based ETLs and their adoption in PSC devices.

## Supporting Information Available

Spectroscopic ellipsometry fits. JV curves, and summary of performance, of reference devices and champion devices. Absorption coefficient spectrum. Composition of SnO_2_, as measured by XPS. FTIR spectra of perovskite/C60 and perovskite/PCBM when exposed to multiple TDMA-Sn doses, or to multiple H_2_O doses, and to 200 ALD SnO_2_ cycles. Integrated area of the incorporated TDMA-Sn ligands. Thickness evolution, measured by SE, of ALD SnO_2_ (PDF)

## References

[ref1] Seo S.; Jeong S.; Park H.; Shin H.; Park N. Atomic layer deposition for efficient and stable perovskite solar cells. Chem. Commun. 2019, 55 (17), 2403–2416. 10.1039/C8CC09578G. 30719523

[ref2] Li H.; Liu M.; Li M.; Park H.; Mathews N.; Qi Y.; Zhang X.; Bolink H. J.; Leo K.; Graetzel M.; Yi C. Applications of vacuum vapor deposition for perovskite solar cells: A progress review. iEnergy 2022, 1 (4), 434–452. 10.23919/IEN.2022.0053.

[ref3] Hossain M. A.; Thong Khoo K.; Cui X.; Poduval G. K.; Zhang T.; Li X.; Li W. M.; Hoex B. Atomic layer deposition enabling higher efficiency solar cells: A review. Nano Mater. Sci. 2020, 2 (3), 204–226. 10.1016/j.nanoms.2019.10.001.

[ref4] Haghighi M.; Ghazyani N.; Mahmoodpour S.; Keshtmand R.; Ghaffari A.; Luo H.; Mohammadpour R.; Taghavinia N.; Abdi-Jalebi M. Low-temperature processing methods for tin oxide as electron transporting layer in scalable perovskite solar cells. Solar RRL 2023, 7 (10), 2201080 10.1002/solr.202201080.

[ref5] George S. M. Atomic layer deposition: an overview. Chem. Rev. 2010, 110 (1), 111–131. 10.1021/cr900056b. 19947596

[ref6] Yu X.; Yan H.; Peng Q. Improve the stability of hybrid halide perovskite via atomic layer deposition on activated phenyl-c61 butyric acid methyl ester. ACS Appl. Mater. Interfaces 2018, 10 (34), 28948–28954. 10.1021/acsami.8b06858. 30058323

[ref7] Brinkmann K.; Zhao J.; Pourdavoud N.; et al. Suppressed decomposition of organometal halide perovskites by impermeable electron-extraction layers in inverted solar cells. Nat. Commun. 2017, 8 (1), 13938 10.1038/ncomms13938. 28067308 PMC5336555

[ref8] Parsons G. N.; Atanasov S. E.; Dandley E. C.; Devine C. K.; Gong B.; Jur J. S.; Lee K.; Oldham C. J.; Peng Q.; Spagnola J. C.; Williams P. S. Mechanisms and reactions during atomic layer deposition on polymers. Coord. Chem. Rev. 2013, 257, 3323–3331. 10.1016/j.ccr.2013.07.001.

[ref9] Yang F.; Brede J.; Ablat H.; Abadia M.; Zhang L.; Rogero C.; Elliott S. D.; Knez M. Reversible and Irreversible Reactions of Trimethylaluminum with Common Organic Functional Groups as a Model for Molecular Layer Deposition and Vapor Phase Infiltration. Adv. Mater. Interfaces 2017, 4 (18), 1700237 10.1002/admi.201700237.

[ref10] Weisbord I.; Shomrat N.; Azoulay R.; Kaushansky A.; Segal-Peretz T. Understanding and Controlling Polymer–Organometallic Precursor Interactions in Sequential Infiltration Synthesis. Chem. Mater. 2020, 32 (11), 4499–4508. 10.1021/acs.chemmater.0c00026.

[ref11] Richey N. E.; de Paula C.; Bent S. F. Understanding chemical and physical mechanisms in atomic layer deposition. J. Chem. Phys. 2020, 152 (4), 040902 10.1063/1.5133390. 32007080

[ref12] Parsons G. N.; Clark R. D. Area-Selective Deposition: Fundamentals, Applications, and Future Outlook. Chem. Mater. 2020, 32 (12), 4920–4953. 10.1021/acs.chemmater.0c00722.

[ref13] Gong B.; Parsons G. N. Quantitative in situ infrared analysis of reactions between trimethylaluminum and polymers during Al2O3 atomic layer deposition. J. Mater. Chem. 2012, 22 (31), 15672–15682. 10.1039/c2jm32343e.

[ref14] Wang W.; Yang Z.; Ding J.; Kong J.; Li X. Improving water-resistance of inverted flexible perovskite solar cells via tailoring the top electron-selective layers. Sol. Energy Mater. Sol. Cells 2022, 238, 111609 10.1016/j.solmat.2022.111609.

[ref15] Raiford J. A.; Boyd C. C.; Palmstrom A. F.; Wolf E. J.; Fearon B. A.; Berry J. J.; McGehee M. D.; Bent S. F. Enhanced nucleation of atomic layer deposited contacts improves operational stability of perovskite solar cells in air. Adv. Energy Mater. 2019, 9 (47), 1902353 10.1002/aenm.201902353.

[ref16] Palmstrom A. F.; Eperon G. E.; Leijtens T.; Prasanna R.; Habisreutinger S. N.; Nemeth W.; Gaulding E. A.; Dunfield S. P.; Reese M.; Nanayakkara S.; Moot T.; Werner J.; Liu J.; To B.; Christensen S. T.; McGehee M. D.; van Hest M. F. A. M.; Luther J. M.; Berry J. J.; Moore D. T. Joule 2019, 3 (9), 2193–2204. 10.1016/j.joule.2019.05.009.

[ref17] Heil S. B. S.; Van Hemmen J. L.; Hodson C. J.; Singh N.; et al. Deposition of Tin and HfO2 in a Commercial 200 mm Remote Plasma Atomic Layer Deposition Reactor. J. Vac. Sci. Technol. A 2007, 25 (5), 1357–1366. 10.1116/1.2753846.

[ref18] Merkx M. J. M.; Sandoval T. E.; Hausmann D. M.; Kessels W. M. M.; Mackus A. J. M. Mechanism of Precursor Blocking by Acetylacetone Inhibitor Molecules During Area-Selective Atomic Layer Deposition of SiO2. Chem. Mater. 2020, 32 (8), 3335–3345. 10.1021/acs.chemmater.9b02992.

[ref19] Mishra S.; Nguyen H. Q.; Huang Q. R.; Lin C. K.; Kuo J. L.; Patwari G. N. Vibrational Spectroscopic Signatures of Hydrogen Bond Induced NH Stretch-Bend Fermi-Resonance in Amines: The Methylamine Clusters and Other N-H···N Hydrogen-Bonded Complexes. J. Chem. Phys. 2020, 153 (19), 194301 10.1063/5.0025778. 33218240

[ref20] Correa-Baena J. P.; Saliba M.; Buonassisi T.; Gratzel M.; Abate A.; Tress W.; Hagfeldt A. Promises and Challenges of Perovskite Solar Cells. Science 2017, 358 (6364), 739–744. 10.1126/science.aam6323. 29123060

[ref21] www.detect99.nl. (accessed on Jan 1, 2024).

[ref22] Barradas N.; Jeynes C. Advanced physics and algorithms in the IBA Data Furnace. Nucl. Instr. and Meth. B 2008, 266 (8), 1875–1879. 10.1016/j.nimb.2007.10.044.

[ref23] Gil-Escrig L.; Dreessen C.; Palazon F.; Hawash Z.; Moons E.; Albrecht S.; Sessolo M.; Bolink H. J. Efficient Wide-Bandgap Mixed-Cation and Mixed-Halide Perovskite Solar Cells by Vacuum Deposition. ACS Energy Lett. 2021, 6 (2), 827–836. 10.1021/acsenergylett.0c02445. 34568574 PMC8461651

[ref24] Raninga R. D.; Jagt R. A.; Bechu S.; Huq T. N.; Li W.; Nikolka M.; Lin Y.; Sun M.; Li Z.; Li W.; et al. Strong performance enhancement in lead-halide perovskite solar cells through rapid, atmospheric deposition of n-type buffer layer oxides. Nano Energy 2020, 75, 104946 10.1016/j.nanoen.2020.104946.

[ref25] Kuang Y.; Zardetto V.; van Gils R.; Karwal S.; Koushik D.; Verheijen M. A.; Black L. E.; Weijtens C. H. L.; Veenstra S.; Andriessen R.; et al. Low-temperature plasma-assisted atomic-layer-deposited SnO_2_ as an electron transport layer in planar perovskite solar cells. ACS Appl. Mater. Interfaces 2018, 10 (36), 30367–30378. 10.1021/acsami.8b09515. 30113160 PMC6137428

[ref26] Mullings M. N.; Hagglund C.; Bent S. F. Tin oxide atomic layer deposition from tetrakis (dimethylamino) tin and water. J. Vacuum Sci. Technol. A 2013, 31 (6), 061503 10.1116/1.4812717.

[ref27] Hu T.; Becker T.; Pourdavoud N.; Zhao J.; Brinkmann K. O.; Heiderhoff R.; Gahlmann T.; Huang Z.; Olthof S.; Meerholz K.; et al. Indium-free perovskite solar cells enabled by impermeable tin-oxide electron extraction layers. Adv. Mater. 2017, 29 (27), 1606656 10.1002/adma.201606656. 28481051

[ref28] Atay F.; Bilgin V.; Akyuz I.; Ketenci E.; Kose S. Optical characterization of SnO_2_: F films by spectroscopic ellipsometry. J. Non-Cryst. Solids 2010, 356 (41–42), 2192–2197. 10.1016/j.jnoncrysol.2010.07.007.

[ref29] Kim D.; Kim D. H.; Riu D.; Choi B. J. Temperature effect on the growth rate and physical characteristics of SnO_2_ thin films grown by atomic layer deposition. Arch. Metall. Mater. 2018, 63 (2), 1061–1064.

[ref30] Choi M.-J.; Cho C. J.; Kim K.; Pyeon J. J.; Park H.; Kim H.; Han J. H.; Kim C. J.; Chung T.; Park T. J.; Kwon B.; Jeong D. S.; Baek S.; Kang C.; Kim J.; Kim S. K. SnO_2_ thin films grown by atomic layer deposition using a novel Sn precursor. Appl. Surf. Sci. 2014, 320, 188–194. 10.1016/j.apsusc.2014.09.054.

[ref31] Heo J.; Kim S. B.; Gordon R. G. Atomic layer deposition of tin oxide with nitric oxide as an oxidant gas. J. Mater. Chem. 2012, 22 (11), 4599–4602. 10.1039/c2jm16557k.

[ref32] Bracesco A. E. A.; Jansen J. W. P.; Xue H.; Zardetto V.; Brocks G.; Kessels W. M. M.; Tao S.; Creatore M. In Situ IR Spectroscopy Studies of Atomic Layer-Deposited SnO_2_ on Formamidinium-Based Lead Halide Perovskite. ACS Appl. Mater. Interfaces 2023, 15 (31), 38018–38028. 10.1021/acsami.3c05647. 37501654 PMC10416150

[ref33] Puurunen R. L.; Vandervorst W. Island growth as a growth mode in atomic layer deposition: A phenomenological model. J. Appl. Phys. 2004, 96 (12), 7686–7695. 10.1063/1.1810193.

[ref34] Vervuurt R. H. J.; Kessels W. M. M.; Bol A. A. Atomic Layer Deposition for Graphene Device Integration. Adv. Mater. Interfaces 2017, 4 (18), 2196–7350. 10.1002/admi.201700232.

[ref35] Mazza M. F.; Cabán-Acevedo M.; Fu H. J.; Meier M. C.; Thompson A. C.; Ifkovits Z. P.; Carim A. I.; Lewis N. S. Selective-Area, Water-Free Atomic Layer Deposition of Metal Oxides on Graphene Defects. ACS Materials Au 2022, 2 (2), 74–78. 10.1021/acsmaterialsau.1c00049. 36855765 PMC9888651

[ref36] Gong J. R. Graphene - Synthesis, Characterization, Properties and Applications, 2011.

[ref37] Solanki A.; Tavakoli M. M.; Xu Q.; Dintakurti S. S. H.; Lim S. S.; Bagui A.; Hanna J. V.; Kong J.; Sum T. C. Heavy Water Additive in Formamidinium: A Novel Approach to Enhance Perovskite Solar Cell Efficiency. Adv. Mater. 2020, 32 (23), 1907864 10.1002/adma.201907864. 32350935

[ref38] Hills-Kimball K.; Nagaoka Y.; Cao C.; Chaykovsky E.; Chen O. Synthesis of Formamidinium Lead Halide Perovskite Nanocrystals through Solid-Liquid-Solid Cation Exchange. J. Mater. Chem. C 2017, 5 (23), 5680–5684. 10.1039/C7TC00598A.

[ref39] Wang P.; Guan J.; Galeschuk D. T. K.; Yao Y.; He C. F.; Jing S.; Zhang S.; Liu Y.; Jin M.; Jin C.; Song S. Pressure Induced Polymorphic, Optical and Electronic Transitions of Formamidinium Lead Iodide Perovskite. J. Phys. Chem. Lett. 2017, 8 (10), 2119–2125. 10.1021/acs.jpclett.7b00665. 28440079

[ref40] Dimesso L.; Quintilla A.; Kim Y.-M.; Lemmer U.; Jaegermann W. Investigation of Formamidinium and Guanidinium Lead Tri-Iodide Powders as Precursors for Solar Cells. Mater. Sci. Eng., B 2016, 204, 27–33. 10.1016/j.mseb.2015.11.006.

[ref41] Kalonga G.; Chinyama G. K.; Munyati M. O.; Maaza M. Characterization and optimization of poly (3-hexylthiophene-2, 5- diyl) (P3HT) and [6, 6] phenyl-C61-butyric acid methyl ester (PCBM) blends for optical absorption. J. Chem. Eng. Mater. Sci. 2013, 4 (7), 93–102. 10.5897/JCEMS2013.0148.

[ref42] Yoo S. H.; Kum J. M.; Cho S. O. Tuning the electronic band structure of PCBM by electron irradiation. Nanoscale Res. Lett. 2011, 6, 545 10.1186/1556-276X-6-545. 21970617 PMC3212083

[ref43] Menéndez J.; Page J. B. Vibrational Spectroscopy of C60, Light Scattering in Solids VIII: Fullerenes, Semiconductor Surfaces. Coherent Phonons 2000, 76, 27–95. 10.1007/BFb0084240.

[ref44] Nikonova R. M.; Lad’yanov V. I.; Rekhviashvili S. S.; Pskhu A. V. Thermal Stability of C60 and C70 fullerites. High Temp. 2021, 59, 179–183. 10.1134/S0018151X21020103.

[ref45] Sundar C. S.; Bharathi A.; Hariharan Y.; Janaki J.; Sankara Sastry V.; Radhakrishnan T. S. Thermal decomposition of C60. Solid State Commun. 1992, 84 (8), 823–826. 10.1016/0038-1098(92)90098-T.

[ref46] Vassallo A. M.; Pang L. S. K.; Cole-Clarke P. A.; Wilson M. A. Emission FTIR study of C60 thermal stability and oxidation. J. Am. Chem. Soc. 1991, 113 (20), 7820–7821. 10.1021/ja00020a086.

[ref47] Stetzer M. R.; Heiney P. A.; Fischer J. E.; McGhie A. R. Thermal stability of solid C60. Phys. Rev. B 1997, 55 (1), 127 10.1103/PhysRevB.55.127.

[ref48] Larson B. W.; Whitaker J. B.; Popov A. A.; Kopidakis N.; Rumbles G.; Boltalina O. V.; Strauss S. H. Thermal [6,6] → [6,6] Isomerization and Decomposition of PCBM (Phenyl-C61-butyric Acid Methyl Ester). Chem. Mater. 2014, 26 (7), 2361–2367. 10.1021/cm500594u.

[ref49] Pont S.; Foglia F.; Higgins A. M.; Durrant J. R.; Cabral J. T. Stability of Polymer: PCBM Thin Films under Competitive Illumination and Thermal Stress. Adv. Funct. Mater. 2018, 28 (40), 1802520 10.1002/adfm.201802520.

[ref50] Ma L.; Guo D.; Li M.; Wang C.; Zhou Z.; Zhao X.; Zhang F.; Ao Z.; Nie Z. Temperature-Dependent Thermal Decomposition Pathway of Organic–Inorganic Halide Perovskite Materials. Chem. Mater. 2019, 31 (20), 8515–8522. 10.1021/acs.chemmater.9b03190.

[ref51] Luongo A.; Brunetti B.; Vecchio Ciprioti S.; Ciccioli A.; Latini A. Thermodynamic and Kinetic Aspects of Formamidinium Lead Iodide Thermal Decomposition. J. Phys. Chem. C 2021, 125 (40), 21851–21861. 10.1021/acs.jpcc.1c06729. PMC8521522 34676017

[ref52] Zheng Z.; Wang S.; Hu Y.; Rong Y.; Mei A.; Han H. Development of formamidinium lead iodide-based perovskite solar cells: efficiency and stability. Chem. Sci. 2022, 13 (8), 2167–2183. 10.1039/D1SC04769H. 35310498 PMC8865136

[ref53] Juarez-Perez E. J.; Ono L. K.; Qi Y. Thermal degradation of formamidinium based lead halide perovskites into sym-triazine and hydrogen cyanide observed by coupled thermogravimetry-mass spectrometry analysis. J. Mater. Chem. A 2019, 7 (28), 16912–16919. 10.1039/C9TA06058H.

[ref54] Pool V. L.; Dou B.; Van Campen D.; et al. Thermal engineering of FAPbI3 perovskite material via radiative thermal annealing and in situ XRD. Nat. Commun. 2017, 8 (1), 14075 10.1038/ncomms14075. 28094249 PMC5247577

