# Supplementary material for: In Situ IR Spectroscopy Studies of Atomic Layer-Deposited SnO2 on Fullerenes for Perovskite Photovoltaics
Source: ACS Appl Mater Interfaces. 2024 Oct 16;16(43):59468–76. doi: 10.1021/acsami.4c09630 (PMC11533177; doi:10.1021/acsami.4c09630)
Supplement: Supplementary file 1 — am4c09630_si_001.pdf [file am4c09630_si_001.pdf]

Supporting Information

In-situ IR spectroscopy studies of atomic layer deposited SnO_2 on fullerenes for perovskite photovoltaics

Andrea E.A. Bracesco[1], Joost van Himste[1], Wilhelmus M.M. Kessels[1], Valerio Zardetto[2], Mariadriana Creatore[1,3,]*

[1] Plasma & Materials Processing, Department of Applied Physics and Science Education, Eindhoven University of Technology (TU/e), 5600 MB Eindhoven, The Netherlands

[2] TNO-partner in Solliance, High Tech Campus 21, 5656 AE Eindhoven, The Netherlands

[3] Eindhoven Institute of Renewable Energy Systems (EIRES), 5600 MB Eindhoven, The Netherlands

* *Corresponding author: m.creatore@tue.nl*

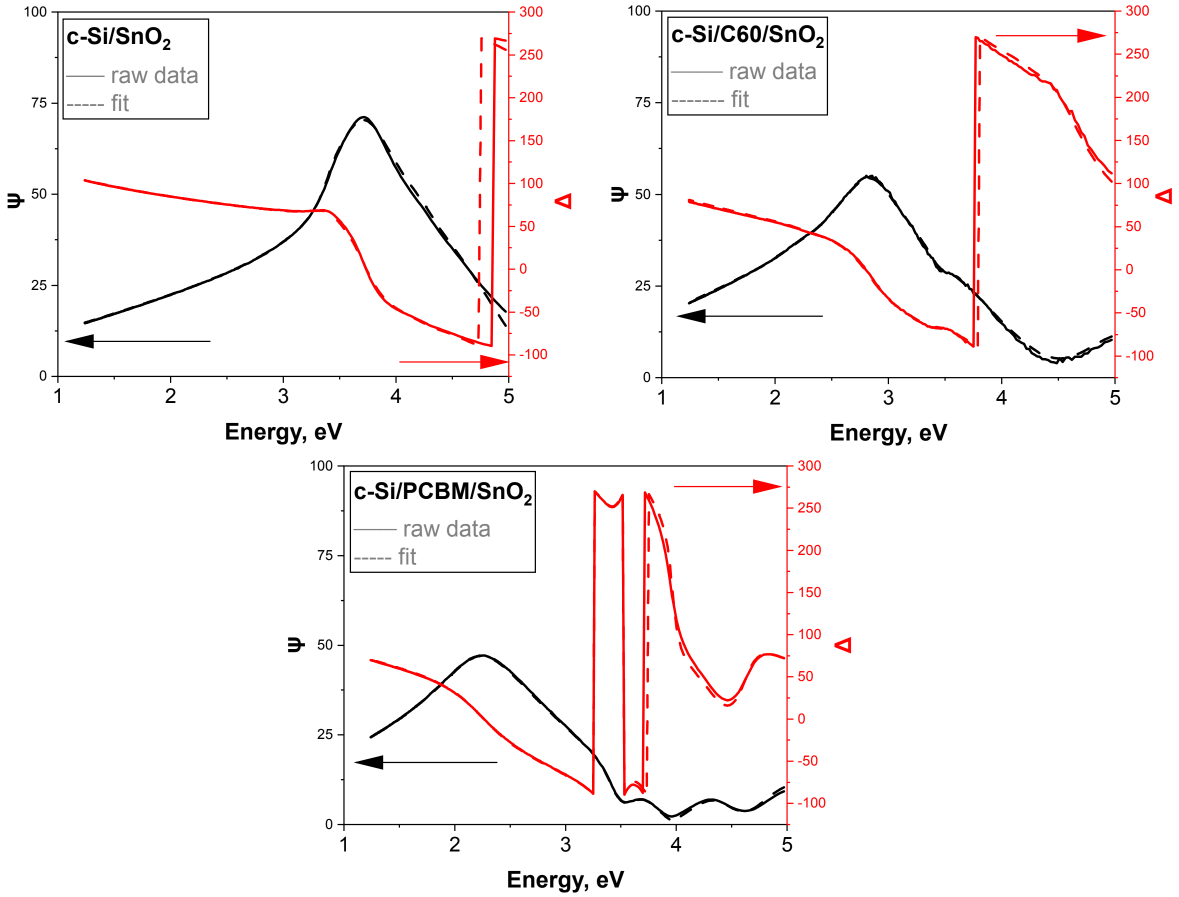

Figure S1: Spectroscopic ellipsometry experimental data and corresponding fits.

Table S1: Fit parameters for the SE data in the 1.2 to 3.1 eV range.

Sample	A (Cauchy)	B (Cauchy)	k amplitude (Urbach)	Exponent (Urbach)	Band Edge (Urbach)
c-Si	1.777	0.024	0.022	1.455	3.1 eV
c-Si/PCBM	1.78	0.025	0.023	1.526	3.1 eV
c-Si/C60	1.788	0.023	0.045	1.125	3.1eV

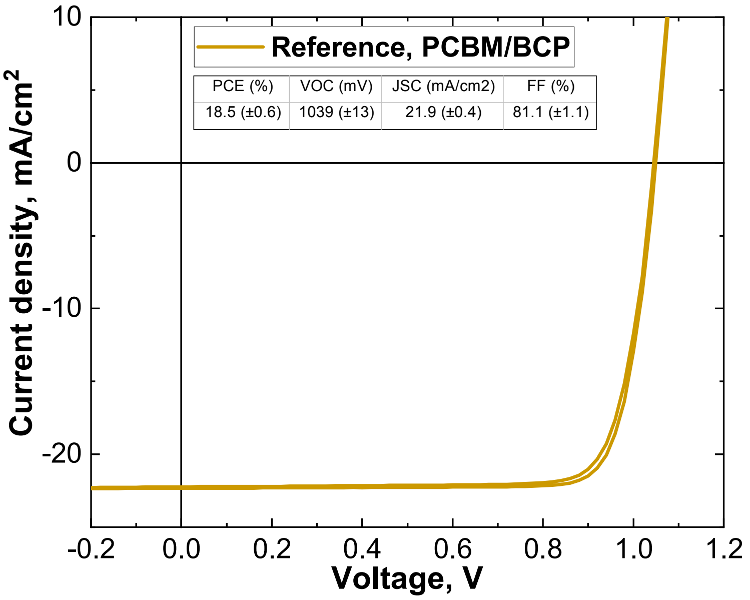

Figure S2: JV curve of the champion PSC device employing a PCBM/BCP ETL. In the insert the JV parameters of the champion device are given.

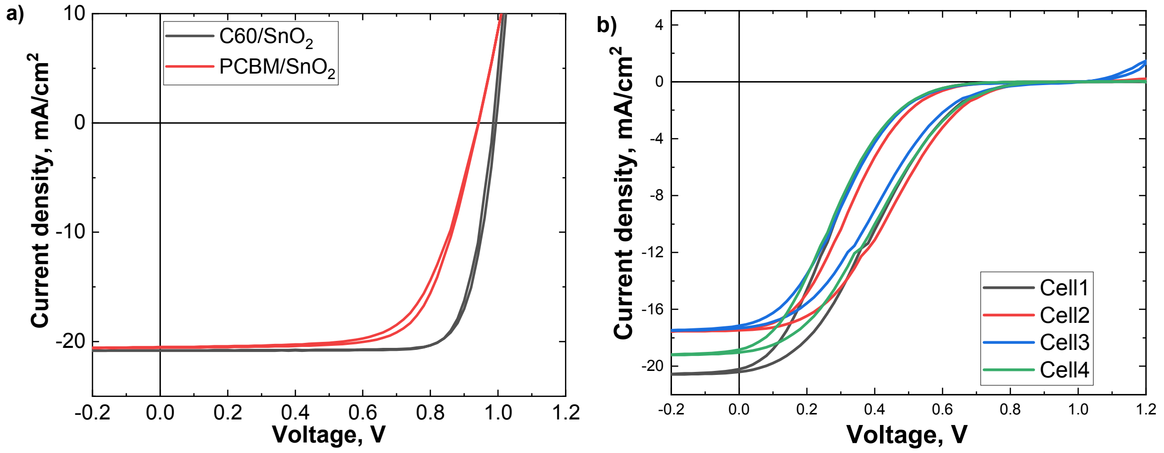

Figure S3: *a) JV curves of the champion devices employing the following ETL stacks: PCBM/SnO_2, and C60/SnO_2. The ALD SnO_2 layer thickness was estimated to be 48 nm. b) JV curves measured on devices employing C60/ SnO_2, where the SnO_2 thickness (12 nm) was not sufficient to prevent ITO sputtering damage of the underlying layers.*

Table SII: Summary of measured JV characteristics of the processed devices: PCE, JSC, FF, and V_{OC}. The device stack for all samples was: glass/ITO/PTAA/perovskite/ETL. Reported are the ETL variations examined.

Device stack	PCE (%)	JSC (mA/cm^2)	FF (%)	VOC (mV)
PCBM/BCP/Cu	18.5(±0.5)	21.8(±0.1)	81.9(±0.8)	1035(±8)
PCBM/SnO_2/ITO/Ag	13.4(±0.4)	20.4(±0.2)	69.6(±3)	947(±20)
C60/SnO_2/ITO/Ag	16.3(±0.3)	20.8(±0.1)	79.7(±1.4)	988(±17)

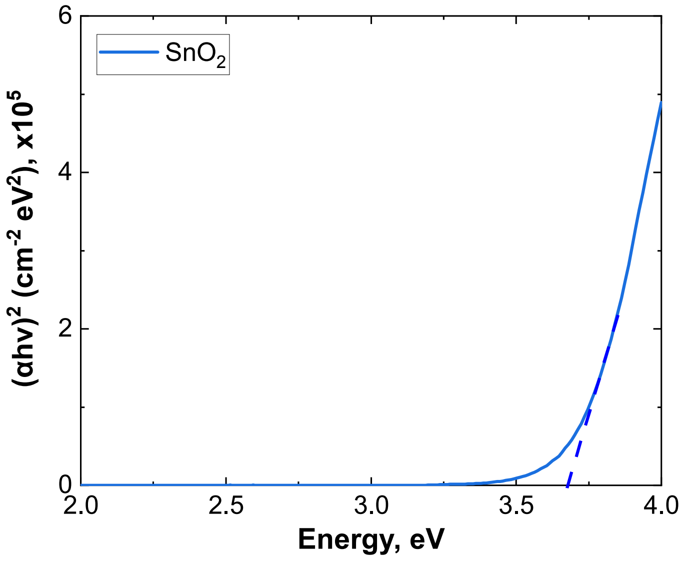

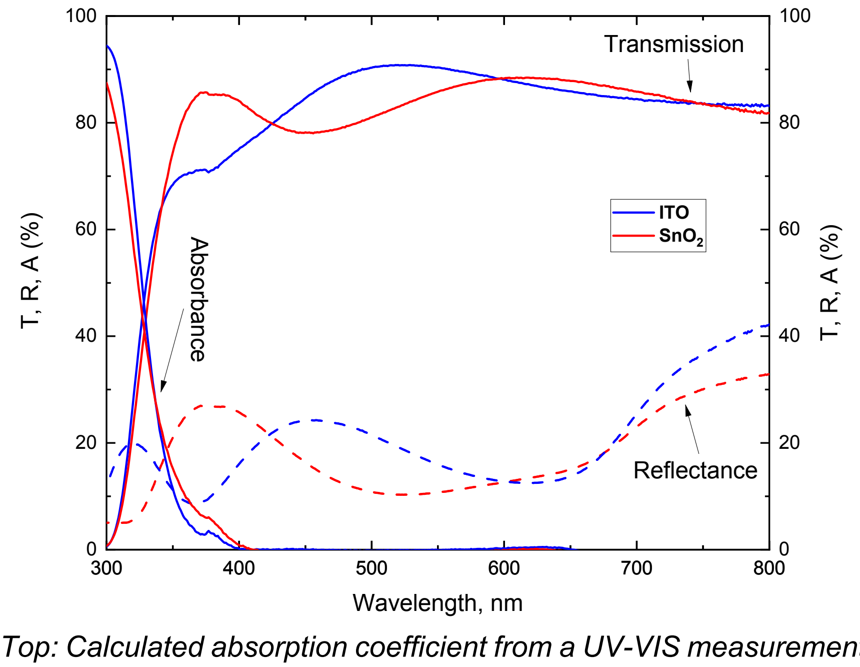

***Figure S4:** Top: Calculated absorption coefficient from a UV-VIS measurement of SnO_2 grown on glass/ITO substrate. The dotted line is the extrapolation of the linear region of the absorption coefficient and its intersect with the axis corresponds to an estimated direct band-gap of 3.60±0.05 eV. Bottom: Measured transmittance, reflectance, and absorbance of the bare glass/ITO substrate and decorated with ALD SnO_2.*

Table SIII: Composition of SnO_2 layers measured by XPS.

Sample	C(at%) ±0.7%	N(at%) ±0.2%	O(at%) ±0.9%	Sn(at%) ±0.4%	O:Sn ±0.2%
c-Si/SnO_2	6.5	2.5	56.6	33.5	2.3
C60/SnO_2	5.8	2.1	57.1	34	2.4
PCBM/SnO_2	6.2	1.4	61.2	35.1	2.4

**The XPS measurements are carried out on c-Si substrates. The O:Sn at. ratios are calculated considering both the oxygen species bound to Sn, as well as the oxygen present as hydroxyl groups.*

Table SIV: Composition and mass density of ALD SnO_2 layers measured by RBS

Sample	C(at/cm²)	N(at/cm²)	O(at/cm²)	Sn(at/cm²)	H(at/cm²)	O:Sn	g/cm³
C60/SnO_2	33.3 (± 0.1)	10.5 (± 0.2)	146 (± 3)	73.3 (± 0.6)	113 (± 3)	2.0 ±0.2%	4.6 (± 0.4)
PCBM/SnO_2	19.2 (± 0.2)	3.4 (± 0.1)	38.5 (± 0.7)	21.5 (± 0.1)	37.4 (± 1)	1.8 ±0.2%	4.3 (± 0.6)

***The RBS/ERD results are measured on a glassy carbon/Al_2O_3 substrate and the values are reported as 10^{15} at/cm². The density is calculated considering the respective SnO_2 layer thickness measured by SE.*

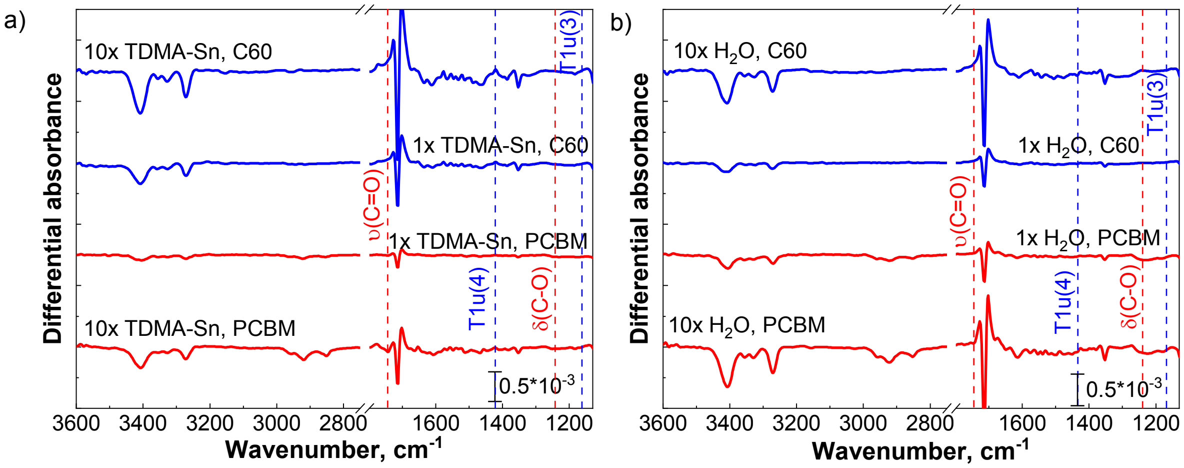

Figure S5: *Differential absorbance spectra (with respect to the pristine perovskite spectrum shown in Figure 5a of: a) the effect of the exposure of perovskite/C60 and perovskite/PCBM to 1 and 10 TDMA-Sn doses and b) the effect of the exposure of perovskite/C60 and perovskite/PCBM to 1 and 10 H_2O doses. The unassigned features correspond to vibrational modes belonging to the perovskite.*

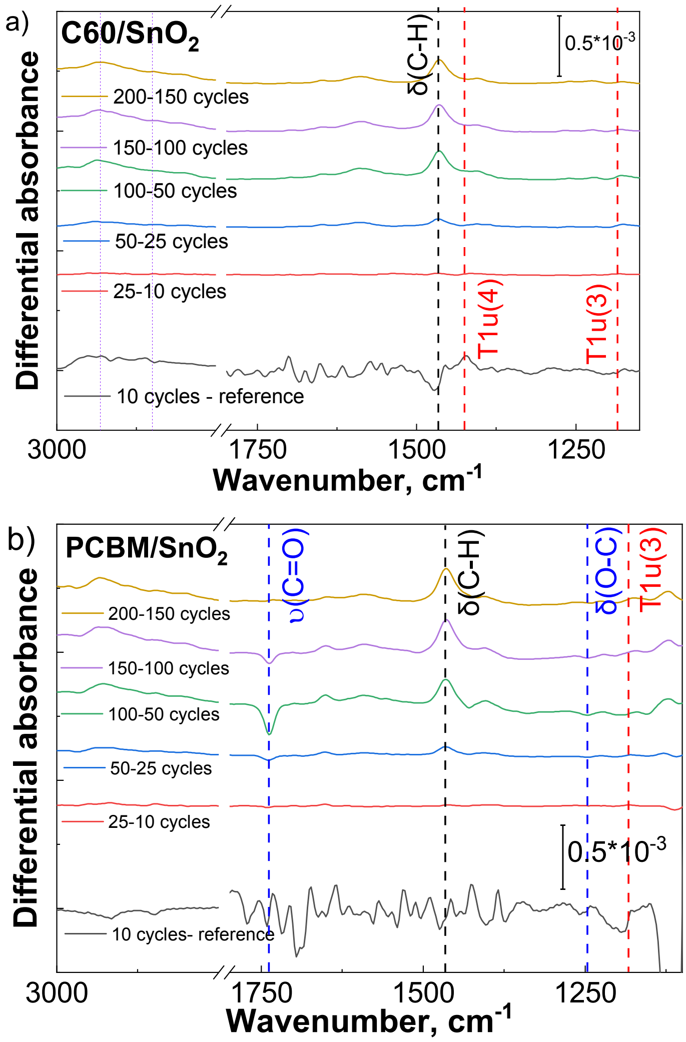

Figure S6: *a) Differential absorbance spectra of a) c-Si/C60 and b) c-Si/PCBM exposed to subsequent numbers of ALD cycles (10-200).*

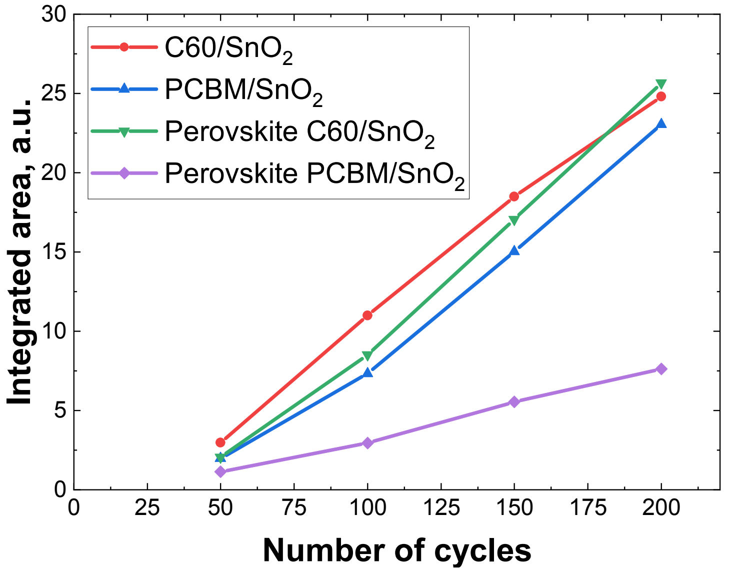

Figure S7: *Evolution of the calculated integrated area of the absorption band at 1466 cm*$^{-1}$*, corresponding to CH*$_x$ *bending mode, originated by the inclusion of TDMA-Sn ligands.*

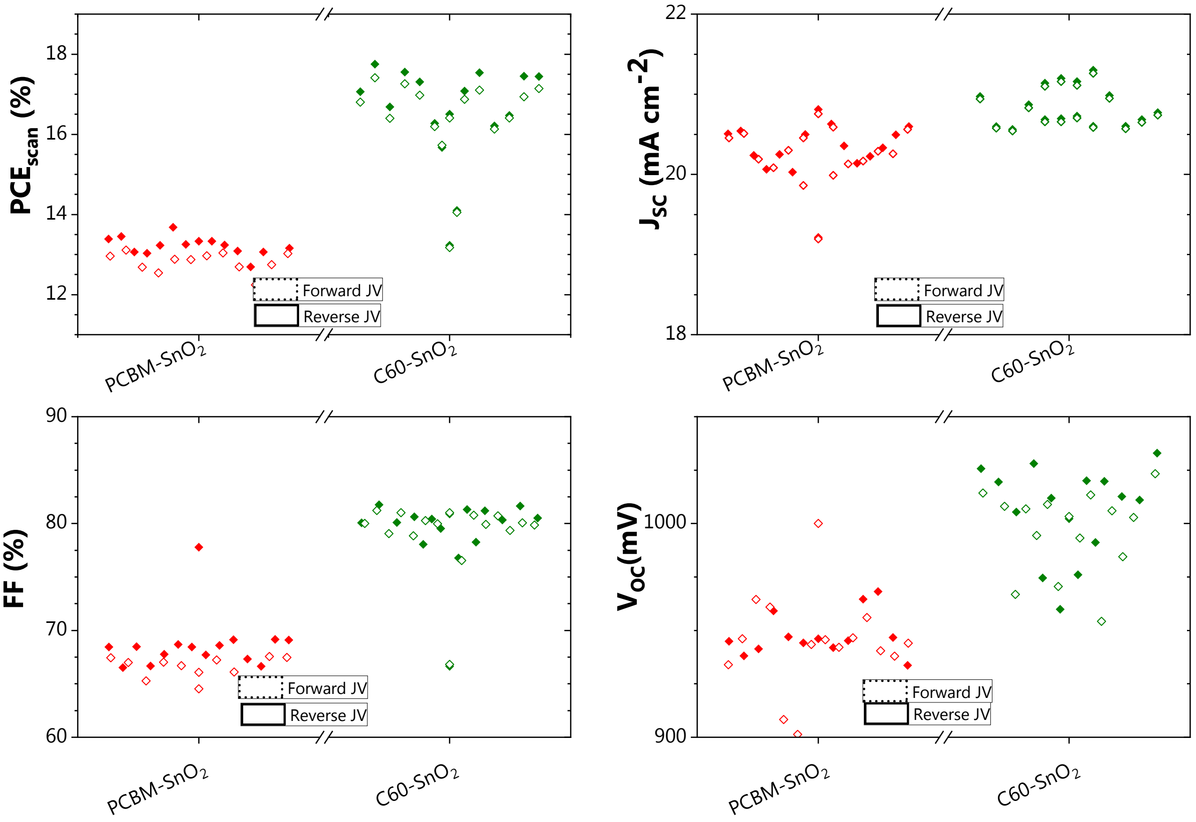

Figure S8: *JV parameters, PCE, Jsc, FF and V*$_{oc}$ *of the reference PSC devices employing a spatial ALD SnO*$_2$ *buffer layer.*

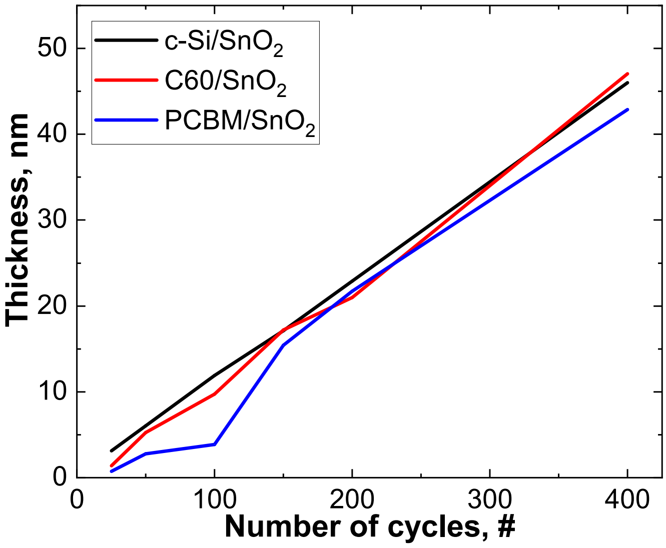

Figure S9: *Thickness evolution of spatial ALD SnO_2 on c-Si, C60, and PCBM, measured by ex situ SE, every 10 cycles.*
